# The Impact of Breast Reduction Surgery on Mental Health and Well-Being: A Systematic Review

**DOI:** 10.1093/asjof/ojag067

**Published:** 2026-04-16

**Authors:** Faris Aldaghri

## Abstract

Breast reduction (reduction mammaplasty) aims to enhance physical appearance and body function improvement. Beyond its cosmetic purpose, growing evidence suggests that these interventions significantly influence psychological health, self-esteem, and overall well-being. However, concerns remain regarding the ethical, psychosocial, and medical implications of such procedures. This systematic review aimed to evaluate the impact of breast reduction on the psychological health, body image, self-esteem, sexual function, and overall quality of life of women undergoing these procedures for purely cosmetic reasons. Following PRISMA (Preferred Reporting Items for Systematic Reviews and Meta-Analyses) guidelines, a comprehensive search was conducted across PubMed, Embase, and Cochrane Library up to September 2025. Eligible studies included adult females (≥18 years) who underwent breast reduction surgery primarily for cosmetic purposes. Studies assessing psychological, psychosocial, or quality-of-life outcomes were included, whereas those involving breast reconstruction for cancer or noncosmetic indications were excluded. Data extraction and quality assessment were independently performed by 2 reviewers using the ROB 2.0 tool. Out of 442 records identified, 7 studies met the inclusion criteria. The findings consistently demonstrated that reduction mammaplasty led to significant improvements in self-esteem, body image, and mental health, with marked reductions in depression and anxiety levels (*P* < .05 across studies). Patients reported enhanced sexual satisfaction and function, as well as substantial gains in quality of life. Physical benefits such as pain relief and increased functional capacity further reinforced these psychological gains. Reduction mammaplasty produces meaningful psychological and quality-of-life benefits in women with body dissatisfaction or symptomatic hypertrophy. Improvements in body image, self-esteem, and mental well-being are consistently reported across studies. However, these benefits must be balanced against surgical risks and ethical considerations, emphasizing the need for comprehensive preoperative psychological evaluation and counseling.

Level of Evidence: 3 (Therapeutic)

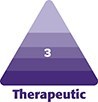

The desire to alter breast size is a complex and multifactorial phenomenon, influenced by factors such as societal beauty ideals, self-confidence, self-esteem, and body image.^1-6^ These elements collectively motivate women across various age groups to pursue plastic or aesthetic breast surgery. Body image refers to an individual's perception and representation of their own physical appearance, shaped by an interplay of perceptual and neural mechanisms, environmental influences, social interactions, and psychological factors. It is reflected in the level of care, attention, and satisfaction a person feels toward their own body.^6^ Excess breast density and skin laxity can limit physical activity and reduce women's work ability and overall productivity.^7-9^ These anxieties can manifest as avoidance of external, social, or intimate situations, which contribute to low self-esteem, anxiety, and even depression. Women affected by breast hyperplasia often report difficulty finding properly fitting clothes and often withdraw from social interactions because of feelings of inadequacy.^10-14^

Despite the growing popularity and purported psychological benefits of aesthetic breast surgery, many objections and concerns have been raised regarding its necessity, safety, and ethical implications. Critics argue that such procedures can reinforce unrealistic beauty standards and perpetuate body dissatisfaction, especially among women influenced by media portrayals and social pressure. There are also concerns that reported psychological improvements after surgery may be temporary or superficial, failing to address underlying self-esteem or mental health issues. Ethical concerns are heightened when psychological vulnerability or physical pathological tendencies drive the decision to undergo surgery. Thus, although breast reduction may increase physical satisfaction for some individuals, its psychological, ethical, and medical implications require careful consideration and thorough preoperative counseling. The aim of this review is to evaluate in detail the psychological impact of reduction mammaplasty.

## METHODS

The methodology followed the PRISMA (Preferred Reporting Items for Systematic Reviews and Meta-Analyses) guidelines to ensure scientific rigor, reproducibility, and transparency.^15^

### Protocol and Registration

The review protocol was not registered in PROSPERO or any other public registry. Because the review was conducted using data from previously published literature, ethical approval was not required.

### Eligibility Criteria

Eligibility was determined using the PICOS (Population, Intervention, Comparison, Outcome, and Study Design) framework.

### Inclusion Criteria

1. Adult female patients (≥18 years) undergoing breast reduction.
2. Studies assessing psychological or psychosocial outcomes postsurgery.
3. Any aesthetic breast surgery performed primarily for cosmetic enhancement or appearance-related concerns, rather than for reconstructive or oncological purposes.
4. Randomized controlled trials (RCTs), cohort studies, case–control studies, and cross-sectional studies.
5. Both prospective and retrospective observational studies.
6. Studies published in English.
7. Peer-reviewed journal articles.

### Exclusion Criteria

- Patients undergoing breast reconstruction after mastectomy or lumpectomy for breast cancer or other oncologic indications.
- Studies not reporting quantitative or qualitative psychological outcomes.
- Studies focusing solely on surgical technique, aesthetic outcomes, or complication rates without psychological assessment.
- Case reports, case series with <10 participants, letters, editorials, commentaries, reviews, or conference abstracts without full data.
- Non-English publications.
- Unpublished theses, dissertations, or gray literature (unless containing extractable and peer-reviewed data).

### Information Sources and Search Strategy

A comprehensive and systematic literature search was carried out across multiple electronic databases to identify relevant studies. The databases searched included PubMed, Cochrane Library, Embase, and others. The search strategy was carefully constructed using a combination of Medical Subject Headings (MeSH). Boolean operators (AND/OR) were used to build a logical and inclusive query. The following search string represents an example of the final query:

(“Breast” [Mesh] OR breast[tiab]) AND (“Mammaplasty” [Mesh] OR mammaplasty[tiab] OR “breast augmentation” [tiab] OR “breast reduction” [tiab] OR “breast lift” [tiab] OR mastopexy[tiab] OR “breast implant” [tiab] OR “cosmetic breast surgery” [tiab] OR “aesthetic breast surgery” [tiab]) AND (“Cosmetic Techniques” [Mesh] OR “Surgery, Plastic” [Mesh] OR “cosmetic surgery” [tiab] OR “aesthetic surgery” [tiab] OR “plastic surgery” [tiab]) AND (“Mental Health” [Mesh] OR “Psychological Well-Being” [Mesh] OR “Quality of Life” [Mesh] OR “Body Image” [Mesh] OR “Self Concept” [Mesh] OR “Depression” [Mesh] OR “Anxiety” [Mesh] OR “Emotions” [Mesh] OR “psychological” [tiab] OR “mental health” [tiab] OR “psychosocial” [tiab] OR “well-being” [tiab] OR “self-esteem” [tiab] OR “body image” [tiab] OR “depression” [tiab] OR “anxiety” [tiab] OR “life satisfaction” [tiab])

The search was limited to studies published in English from database inception to September 2025.

### Screening of Studies

The studies that were retrieved after the search were then screened in 2 stages. First, the titles and the abstracts of all the identified articles were screened by 2 reviewers to determine their relevance according to the inclusion and exclusion criteria. Some of the studies that were eliminated in this step are those that could not meet the laid-down criteria. If the relevance of the article was not clear, then the whole content of the article was downloaded for further assessment.

In the second phase, full-text articles of the studies that may be related to the current study were screened by the 2 reviewers. In case there were any differences on the eligibility of the study, the same were resolved by reaching a consensus or with the help of a third reviewer. Such a screening process was effective in filtering out low-quality and irrelevant studies to be included in the final analysis. The study selection process and rationale for inclusion/exclusion were transparently documented using a PRISMA flow diagram.^16^

### Data Extraction

A standardized data extraction form was developed and used by 2 reviewers to independently extract data from each included study. The following information was collected:

- Study characteristics, including author names, publication year, study design, and setting.
- Patient demographics, including age, sex, and comorbidities.
- Outcomes and results.

The extracted data were then cross-checked by both reviewers to ensure consistency and accuracy. Any discrepancies were discussed, and a consensus was reached before finalizing the dataset.

### Risk of Bias Assessment

To evaluate the methodological quality of the included studies, the ROB 2.0 was employed for the included studies.^17^ All risk assessments were conducted independently by 2 reviewers. Inconsistencies in ratings were discussed and reconciled with the help of a third reviewer where necessary. The overall quality of papers included adheres to strict methodological standards. However, the interpretation from Moss and Harris and Sabino Neto et al warrants a degree of caution regarding intervention adherence and data completeness. The results of the risk of bias assessments were visually presented using structured summary tables.

## RESULTS

### Study Selection and Screening

**Figure 1.**
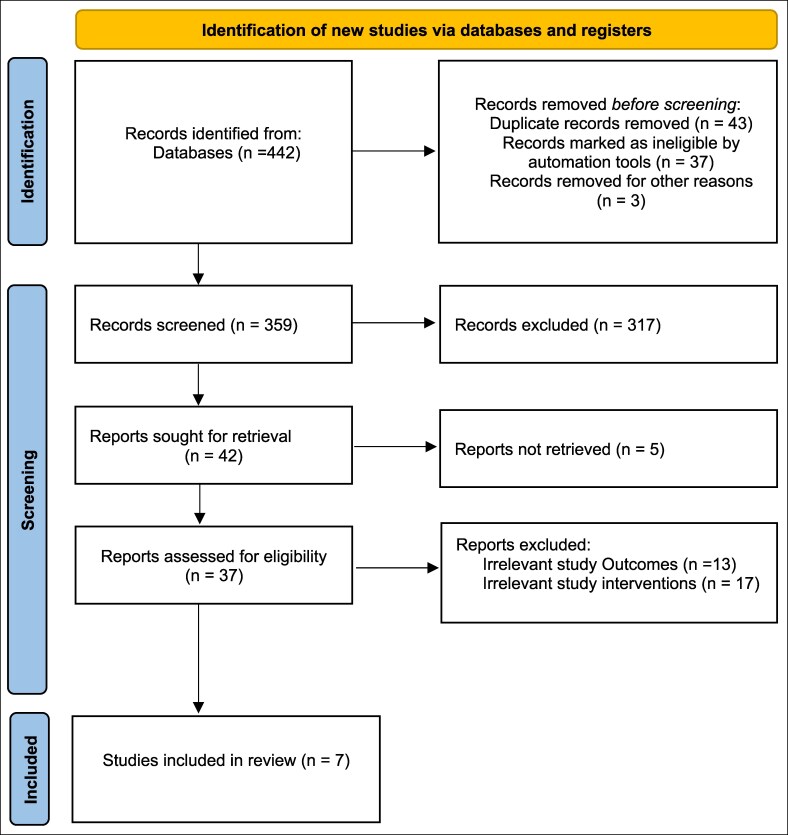
PRISMA flow diagram shows the identification, screening, and inclusion of the articles in the review. Seven studies were included in the final review.

The initial search of the database yielded 442 papers. After the removal of duplicates and applying the inclusion criteria, a total of 37 studies were selected for full-text analysis. Based on the methodological quality assessment and inclusion and exclusion criteria, a total of 7 articles finally met the criteria to be included in this systematic review. Figure 1 presents the detailed PRISMA flowchart diagram of the selection process of the included studies. The characteristics of all the included studies are given in Table 1.

**Table 1.** Characteristics of Included Studies

Author	Year	Study design	Population	Sample size	Surgery type	Outcome measures	Outcomes	Psychological outcomes
Beraldo et al^18^	2016	RCT, prospective	Breast hypertrophy patients	Initial: 60 (CG: *n* = 30, BRG: *n* = 30). Completed 6-month follow-up: 56 (CG: 27, BRG: 29)	Reduction mammaplasty (traditional inverted T-scar medial pedicle technique)	FSFI, BDI	Sexual function and depression outcomes	Depression, sexual satisfaction, orgasm, desire, and excitement (FSFI domains)
Fonseca et al^19^	2018	Prospective evaluation, level of evidence: 2	Women with breast hypertrophy scheduled for reduction mammaplasty (MG) compared with women with normal-sized breasts (NSBG)	MG: 103 women; NSBG: 103 women	Reduction mammaplasty (standardized, conventional, inverted “T” scar)	BDDE, BIS, and Breast Evaluation Questionnaire (BEQ55)	Body image	Concern over physical appearance, negative self-assessment, shame, embarrassment (BDDE), emotional investment in one's body (BIS), symptoms of body dysmorphic disorder
Moss and Harris^20^	2009	Prospective, controlled, quasi-experimental design	Aesthetic surgical patients (breasts, nose, and upper limbs) (PSG) compared with a nonappearance-altering surgical group (CSG; breast operations were mainly reductions)	PSG: 51 (18 breast) CSG: 105 (44 completed T1/T2)	Aesthetic plastic surgery (including breast reductions)	Crown–Crisp Experiential Inventory Anxiety Scale, BDI, DAS24	Anxiety, depression, appearance distress/dysfunction	Anxiety and depression (general), distress and dysfunction related to self-consciousness of appearance (DAS24)
Sabino Neto et al^21^	2008	Prospective study, randomly allocated patients	100 patients with breast hypertrophy	Initial: 100 (Group A [mammaplasty]: 50, Group B [control]: 50). Completed study: 46 in each group	Reduction mammaplasty (inverted-T approach)	Rosenberg Self-Esteem Scale, Roland–Morris questionnaire (functional capacity), Visual Analog Scale (VAS) (low back pain).	Self-esteem, functional capacity, pain relief	Self-esteem
Saariniemi et al (QoL comparison)^22^	2007	Prospective, RCT data used for comparison study	Patients with symptomatic breast hypertrophy	Baseline: 82. Completed 6-month follow-up (operative group): 29 patients	Reduction mammaplasty	15D health-related QoL questionnaire	Health-related QoL, comparison to general population and joint replacement patients.	Depression, distress, mental function, vitality, sexual activity (dimensions of 15D)
Saariniemi et al (RCT, QoL/Pain)^23^	2008	Prospective RCT	Patients with symptomatic breast hypertrophy	Randomized: 82 (40 operative, 42 nonoperative) Completed study: 64 (29 operative, 35 nonoperative)	Reduction mammaplasty	Short Form-36 (SF-36) QoL questionnaire, 15D QoL questionnaire, Finnish Breast-Associated Symptoms, Finnish Pain Questionnaire	QoL, breast-associated symptoms, and pain	SF-36 mental summary score, 15D index score
Saariniemi et al (Depression/Anxiety)^24^	2009	Prospective RCT	Macromastia patients (symptomatic breast hypertrophy)	Randomized: 82 (40 operation, 42 conservative) Completed study: 64 (29 operated, 35 conservative)	Reduction mammaplasty	RBDI questionnaire (Raitasalo's modification of the short form of the BDI)	Depression, anxiety, and self-esteem	Depression, anxiety, and self-esteem scores derived from the RBDI

BDI, Beck Depression Inventory; BDDE, Body Dysmorphic Disorder Examination; BIS, Body Investment Scale; BRG, breast reduction group; CG, control group; CSG, control surgery group; DAS24, Derriford Appearance Scale-24; FSFI, Female Sexual Function Index; MG, mammaplasty group; NSBG, normal-sized breast group; PSG, plastic surgery group; QoL, quality of life; RCT, randomized clinical trial.

The results extracted from the systematic review studies consistently demonstrate that reduction mammaplasty yields significant positive physical and psychological outcomes, particularly in improving body image, self-esteem, and sexual function and in reducing symptoms of depression and anxiety in women with breast hypertrophy. The key results regarding psychological health and related outcomes are summarized below.

#### Depression and Anxiety

Reduction mammaplasty significantly alleviates symptoms of depression and anxiety across multiple studies:

- Reduction in Depression Scores^18^: Compared with the control group (CG), the breast reduction group (BRG) reported better depression scores 6 months postoperatively (*P* = .014). Regarding the total Beck Depression Inventory (BDI) score, the BRG showed significant improvement at 6 months (*P* = .01). Intriguingly, intragroup assessment showed the BRG had higher BDI scores preoperatively, indicating higher depression levels, which decreased significantly at 3 and 6 months postoperatively (*P* < .001). At 6 months, 76% of the BRG showed minimum depression compared with 33% of the CG (*P* = .01).
- Alleviation of Depression and Anxiety^24^: Operated patients had significantly less depression (*P* ≤ .01), and the proportions of depressed (*P* ≤ .01) and anxious (*P* = .04) patients were smaller in the operated group compared with the conservative group at 6 months. Six months after surgery, four-fifths of operated patients showed no signs of depression or anxiety.
- Reduction in General Anxiety^20^: Although both the aesthetic plastic surgery group (PSG) and the control surgery group were less depressed postoperatively (*P* = .04), the improvement in anxiety was significantly greater in the PSG. These improvements in both anxiety and depression were maintained at 12 months postoperation.
- SF-36 Mental Score: In 1 randomized trial, the findings for the mental summary score of the SF-36 were less significant statistically and clinically when comparing the operative and nonoperative groups, although physical quality of life (QoL) improved highly significantly.

#### Body Image, Self-Esteem, and Appearance Distress

Reduction mammaplasty resulted in strong positive changes in how patients perceive their bodies and their self-worth:

- Appearance Distress^20^: Body-site-specific appearance distress/dysfunction (measured by Derriford Appearance Scale-24 [DAS24]) significantly improved for the aesthetic surgical group only. For breast operations, this improvement was apparent in the first 3 months and was maintained at 12 months.
- Improvement in Self-Esteem^21,22^: A decrease in the score of the Rosenberg Self-Esteem Scale for the mammaplasty group (MG) indicated a significant improvement in self-esteem (*P* < .001) at 6 months postsurgery, whereas the CG showed no change. Operated patients also demonstrated better self-esteem (*P* = .03) compared with the conservative group at 6 months.
- Body Image Improvement^19^: The MG showed significant improvement in Body Dysmorphic Disorder Examination (BDDE), Body Investment Scale (BIS), and Breast Evaluation Questionnaire (BEQ55) scores 6 months postoperatively (*P* ≤ .001 for all 3). After surgery, MG patients were as satisfied as or more satisfied (BEQ55 score) than the normal-sized breast group (NSBG), surpassing the NSBG level of satisfaction and body image. The MG group began to invest more in their body (BIS score), reaching levels like the NSBG group.
- Remission of Body Dysmorphic Disorder (BDD) Symptoms^19^: Preoperatively, a high prevalence of negative body image symptoms characterizing BDD was observed in the MG. Six months postsurgery, the intervention promoted the remission of symptoms of BDD in all MG patients.

#### Sexual Function Outcomes

Reduction mammaplasty positively impacted patients' sexual function:

- Improved Total Sexual Function^18^: The BRG reported better sexual function (Female Sexual Function Index [FSFI] total score) compared with the CG at 3 months (*P* = .015) and 6 months (*P* = .009) postoperatively.
- Specific Domain Improvements: At 6 months, significant improvement was seen in the FSFI domains of desire (*P* = .02), excitement (*P* < .001), lubrication (*P* = .05), orgasm (*P* = .01), and satisfaction (*P* = .01).
- Reduced Sexual Dysfunction: The BRG showed a lower frequency of sexual dysfunction (FSFI total score ≤26.55) at both 3 months (*P* = .01) and 6 months (*P* < .001) postoperatively.

#### QoL and Physical Symptoms

**Figure 2.**
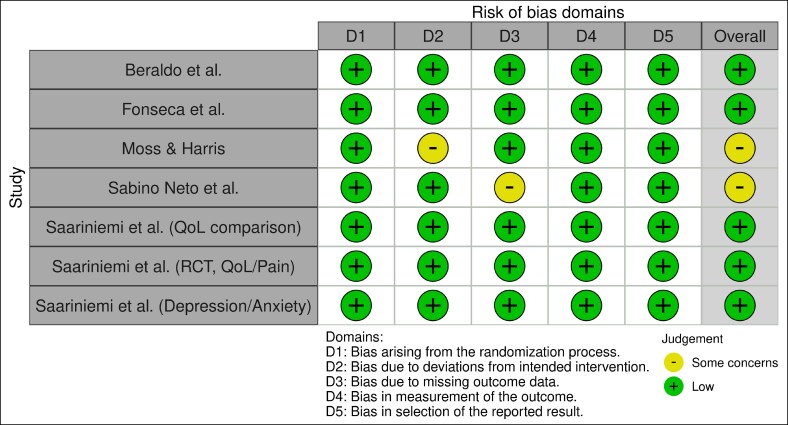
This figure shows the included studies risk of bias with 2 studies that have minimal concern either because of missing outcome data or deviation from intended intervention.

The surgical benefits extended beyond psychological measures to overall QoL and physical relief:

- General QoL Improvement^22^: Preoperatively, patients had significantly lower health-related QoL (15D Index score) than the age-standardized female population. By 6 months postsurgery, QoL improved to the same level as the population.
- Intervention Effect Comparison: The extent of the preoperative health deficit caused by symptomatic breast hypertrophy was equal to that caused by symptomatic major joint arthrosis. The positive effect of the intervention on QoL was comparable to that of hip replacement.
- Functional Capacity and Pain Relief: Functional capacity improved significantly 6 months after reduction mammaplasty. The intensity of low back pain, evaluated by the Visual Analog Scale (VAS), decreased significantly from an average of 5.7 preoperatively to 1.3 postoperatively (*P* < .001). Furthermore, the operative group demonstrated highly significant statistical and clinical differences (*P* < .0001) compared with the nonoperative group for physical QoL, pain (Finnish Breast-Associated Symptoms and Finnish Pain Questionnaire scores), and breast-associated symptoms. The Quality Assessment was done using the ROB 2.0 tool. The traffic light plot is given in Figure 2.

## DISCUSSION

The results from these sources provide strong and consistent evidence that reduction mammaplasty significantly improves the psychological health and QoL of women suffering from breast hypertrophy, demonstrating benefits across specific mental health indicators, body image perception, and sexual function.

The most profound psychological effects documented relate to patients' self-perception and body image adjustment, often elevating the operative group's satisfaction levels to or beyond that of women with normal-sized breasts. Multiple studies confirm the restoration of self-esteem postsurgery. Patients in the reduction MG (Group A) experienced a significant improvement in self-esteem, indicated by a decrease in the Rosenberg Self-Esteem Scale score (*P* < .001). Another randomized trial confirmed that operated patients had better self-esteem (*P* = .03) than the conservative group at 6 months.^18^ Reduction mammaplasty successfully addresses deep dissatisfaction with physical appearance. The MG showed significant improvement in body image scores (BDDE, BIS, and BEQ55) 6 months postoperatively (*P* ≤ .001 for all 3).^19^ Following the procedure, MG patients were found to be as satisfied, or more satisfied, than the NSBG, successfully surpassing the NSBG level of satisfaction and body image. This highlights that the surgery not only corrected the physical anomaly but also promoted a positive overall feeling of well-being in relation to the body. The PSG showed significant improvement in body-site-specific appearance distress (DAS24) compared with the nonappearance-altering surgical group, an improvement that was maintained at 12 months. Crucially, the intervention promoted the remission of symptoms of BDD among patients who had previously exhibited these negative body image symptoms.^22–24^

The studies, particularly those using validated depression scales, noted marked alleviation of depressive and anxious symptoms following surgery. One prospective RCT using the BDI found that the BRG reported better depression scores 6 months postoperatively (*P* = .014) compared with the CG. This study indicated that the BRG initially presented with higher depression levels preoperatively, which significantly decreased at 3 and 6 months postoperatively (*P* < .001). At the 6-month mark, 76% of the BRG showed minimum depression, a significant difference compared with 33% of the CG (*P* = .01). Another RCT using the Revised Beck Depression Inventory (RBDI) found that operated patients had significantly less depression (*P* ≤ .01) and better self-esteem (*P* = .03) than the conservative group at 6 months. The proportions of depressed (*P* ≤ .01) and anxious (*P* = .04) patients were significantly smaller in the operated group. Importantly, 6 months after reduction mammaplasty, four-fifths of operated patients showed no signs of depression or anxiety.^24^ In the controlled quasi-experimental design, although both aesthetic and nonappearance-altering surgical groups experienced a postoperative reduction in depression, the improvement in anxiety was significantly greater in the aesthetic PSG.^20^

Reduction mammaplasty demonstrated a strong positive impact on sexual health, an increasingly recognized aspect of QoL. The BRG reported better sexual function (measured by the FSFI) compared with the CG at 3 months (*P* = .015) and 6 months (*P* = .009) postoperatively. At 6 months postoperatively, significant improvements were seen across multiple FSFI domains, including desire (*P* = .02), excitement (*P* < .001), lubrication (*P* = .05), orgasm (*P* = .01), and satisfaction (*P* = .01). The surgery resulted in a lower frequency of sexual dysfunction (FSFI total score ≤26.55) in the BRG at both 3 and 6 months postoperatively (*P* = .013 and *P* = .003, respectively).^23^

The improvements in psychological outcomes are contextually supported by significant enhancements in physical function and overall health-related QoL. Symptomatic breast hypertrophy causes a considerable health deficit. Preoperatively, patients had significantly lower health-related QoL (15D Index score) than the age-standardized female population. However, reduction mammaplasty completely removed this deficit, and QoL improved to the same level as the population within 6 months. The preoperative health deficit of symptomatic breast hypertrophy was found to be equal to that of symptomatic major joint arthrosis. Consequently, the effect of the reduction mammaplasty intervention on QoL was comparable to that of total hip replacement surgery. The operation dramatically improved physical outcomes, including relieving pain and increasing functional capacity. For instance, low back pain intensity (VAS score) decreased significantly from 5.7 to 1.3 (*P* < .001) in the mammaplasty group.^24^ This great relief of physical symptoms and pain, verified by objective scores, likely underpins the corresponding improvement in psychological aspects. It is important to note that breast augmentation procedures were often evaluated in studies that also included women undergoing reconstructive surgery after breast cancer. Because these mixed populations confound psychological effects because of cosmetic enhancement alone, such studies were excluded from this review. This exclusion ensured that the findings reflected typical outcomes of nononcological, aesthetic breast surgery. However, it also limits conclusions about the psychological effects of increased isolation. Future research should therefore report stratified results separating augmentation from reconstruction to increase clinical applicability and accuracy in understanding patient-reported benefits.

This study also has some limitations. The limited number of eligible studies (*n* = 7) and small sample sizes in several trials reduce the generalizability of the results. The existence of similar work in the literature is reported, but the scope of this review is not identical. Furthermore, short-term follow-up durations (3-6 months) in many studies limit the understanding of long-term psychological outcomes. The absence of meta-analysis because of heterogeneity in study design, outcome measures, and populations prevents pooled effect size estimation. Potential publication bias and language restriction to English may have led to the exclusion of relevant studies.

To summarize the effectiveness demonstrated by these results, reduction mammaplasty acts as a comprehensive therapeutic intervention. By alleviating the heavy physical burden and restoring function, the subsequent relief allows for improvement of many psychological aspects, that is, self-esteem and emotional well-being.

## CONCLUSIONS

This systematic review provides strong evidence that breast reduction significantly enhances psychological well-being, body image satisfaction, and overall QoL among women with breast-related self-image concerns. These improvements lead to measurable reductions in anxiety, depression, and body dysmorphia symptoms, as well as enhanced sexual function and self-esteem. Nonetheless, psychological benefits must be interpreted alongside surgical risks and ethical considerations. Although this review provides strong evidence supporting the psychological and quality-of-life benefits of aesthetic breast surgery, particularly reduction mammaplasty, it highlights an important research gap: most augmentation studies included reconstruction populations and were thus excluded. The findings highlight the importance of thorough preoperative psychological assessment, patient education, and long-term follow-up to ensure lasting mental health outcomes. Future large-scale, longitudinal studies comparing different aesthetic breast procedures are needed to better define the durability and scope of these psychological benefits.
